# Immature Stages of the Neotropical Butterfly, *Dynamine agacles agacles*

**DOI:** 10.1673/031.012.3701

**Published:** 2012-03-17

**Authors:** Luis Anderson Ribeiro Leite, Mirna Martins Casagrande, Olaf Hermann Hendrik Mielke, André Victor Lucci Freitas

**Affiliations:** ^1^ Departamento de Zoologia; Setor de Ciências Biológicas; Universidade Federal do Paraná; C. P.: 19020; 81531980, Curitiba, Paraná, Brazil; ^2^ Departamento de Biologia Animal and Museu de Zoologia, Instituto de Biologia, Universidade Estadual de Campinas; C. P.: 6109; 13083-970, Campinas, São Paulo, Brazil

**Keywords:** bionomy, chaetotaxy, life cycle, Papilionoidea

## Abstract

The external morphology of the immature stages of *Dynamine agacles agacles* (Dalman, 1823) (Lepidoptera: Nymphalidae: Biblidinae) is described, including photos, drawings, and scanning electron micrographs. Data on the adult and larval behavior are given based on observations in the host plant *Dalechampia triphylla* Lam. (Malpighiales : Euphorbiaceae). The results are compared and discussed with other described species of Biblidinae, allowing to make further observations on the natural history and evolution of *Dynamine*.

## Resumo

A morfologia externa dos imaturos de *Dynamine agacles agacles* (Dalman, 1823) (Lepidoptera: Nymphalidae: Biblidinae) é descrita, incluindo fotos, desenhos e microscopia eletrônica de varredura. Dados sobre o comportamento de adultos e larvas são fornecidos com base em observações na planta hospedeira *Dalechampia triphylla* Lam. (Malpighiales : Euphorbiaceae). Os resultados são comparados e discutidos com outras espécies descritas de Biblidinae, permitindo fazer novas observações sobre a história natural e evolução de *Dynamine*.

## Introduction

Biblidinae is a well defined clade of Nymphalidae, and their members are distributed primarily in the Neotropical region, with some species also present in the Paleotropics (Munroe 1949; Van Son 1979; Ackery 1988). Besides being conspicuous and well represented in most Neotropical biomes, many Biblidinae genera are still poorly known, with early stages described for just a small fraction of taxa in the larger genera (Freitas et al. 1997; Barbosa et al. 2010).

*Dynamine* Hübner, [1819] is the richest within Biblidinae, with 41 described species (Lamas 2004; Brévignon 2008; Willmott and Hall 2010). Nevertheless, general information for most species in this genus is lacking, and is mostly restricted to geographic distribution, partial descriptions of immature stages, and host-plant records (DeVries 1987; Doyle 1979; Neild 1996; Freitas et al. 1997; Beccaloni et al. 2008).

**Figures 1–4.**
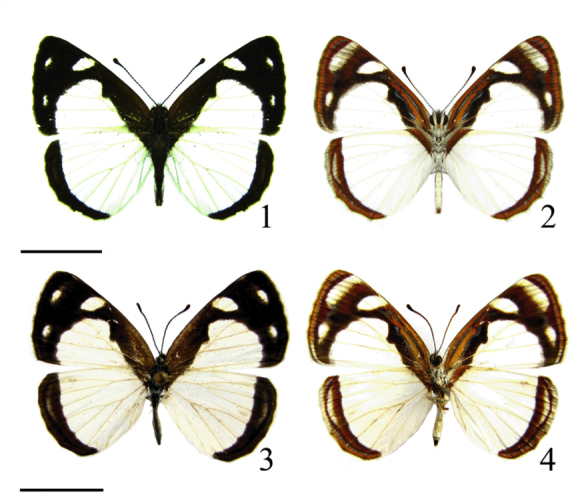
*Dynamine agacles agacles*. (1, 2) Male dorsal and ventral view; (3, 4) Female dorsal and ventral view. Scale bar = 1 cm. High quality figures are available online.

*Dynamine agacles* (Dalman, 1823) (Lepidoptera: Nymphalidae: Biblidinae) is a common species with a range extending from Costa Rica to South Argentina (Neild 1996). According to Lamas (2004), two subspecies are recognized: *Dynamine agacles core* Röber, 1915, occurring from Central America to Venezuela and Colombia, and *Dynamine agacles agacles* (Dalman, 1823), (Figures 1–4), distributed throughout the remainder of South America (DeVries 1987; Neild 1996; Lamas 2004). The known host plants for *D. agacles* are scandent vines in the genus *Dalechampia* (Euphorbiaceae) (DeVries 1987; Beccaloni et al. 2008), and larvae feed on the involucral bracts of mature and developing flowers, and also inside the pseudanthial inflorescences, feeding even in the ovaries, directly affecting flowers and seed production (Neild 1996).

Considering the importance of early stages as sources of characters for establishing phylogenetic hypothesis and for taxonomic studies in higher Lepidoptera taxa (Freitas et al. 1997; Greeney and Gerardo 2001; Hasenfuss and Kristensen 2003; Casagrande and Mielke 2005; Souza et al. 2006; Freitas and Brown Jr. 2004, 2008; Dias et al. 2010a, 2010b), the present study has the objective of presenting detailed morphological and behavioral descriptions of the immature stages of *Dynamine agacles agacles*, aiming to contribute to the knowledge of Neotropical Biblidinae.

## Materials and Methods

Adults and immatures were studied in three localities in Brazil: (1) Parque Barigüi (25° 25′ S, 49° 18′ W, 911 m), (2) Campus of the Universidade Federal do Paraná (25° 26′ S, 49° 13′ W, 919 m), Curitiba, Paraná, South Brazil, and (3) Reserva Florestal da Santa Genebra (22° 49′ S, 47° 6′ W, 650 m, April 1997), Campinas, São Paulo, Southeastern Brazil.

Immatures and the host plant *Dalechampia triphylla* (Euphorbiaceae) were brought to laboratory. Eggs were placed in Petri dishes and observed daily until eclosion. Newly-hatched larvae were reared individually in transparent 250 mL plastic cages. Branches of *D. triphylla* with inflorescences were offered *ad libitum*, and larvae were checked daily for food replacement and cleaning when necessary (or when all bracts were consumed). Images of the eggs and early instars were taken through a digital camera attached to the stereomicroscope using automontage technique, in a Leica® MZ16 (www.leica.com) and software Syncroscopy® Auto-Montage Pro® version 5.03.0040 (www.syncroscopy.com).

Immatures for morphological analysis (at least three of each stage) were killed in boiling water, fixed in Kahle-Dietrich solution, and after three days transferred to 70% ethanol. Shed head capsules and exuviae were collected and preserved dry for measurements. Measurements, drawings, and general aspects of morphology were done in a Leica® MZ16 stereomicroscope equipped with a micrometric scale. Head capsule width of larvae was the distance between the most external stemmata; maximum total length for both larvae and pupae corresponded to the distance from the head to the posterior margin of the tenth abdominal segment in dorsal view (as in Freitas 2007).

Scanning electron microscopy (SEM) was conducted using a Jeol® JSM—6360LV microscope (www.jeol.com). Samples were prepared in accordance with the following protocol: critical point dried in a Bal-tec® CPD030 Critical Point Dryer (www.precisionballs.com) and attached with double stick tape to aluminum stubs; gold/palladium coated with a Bal-tec® SCD030 Sputter Coater.

Terminology for early stage descriptions followed Stehr (1987) for larval structures, Dias (2006) for pupae, both modified after general studies of Lepidoptera morphology (Mosher 1916; Peterson 1962; Casagrande 1979; Scoble 1992; Paim et al. 2004; Duarte et al. 2005; Kaminski et al. 2008; Dias et al. 2010a, 2010b).

Voucher specimens of the immature stages were deposited at the Coleção Entomológica Pe. Jesus Santiago Moure — Lepidoptera of the Universidade Federal do Paraná, Curitiba, Paraná, Brazil, and Museu de Zoologia “Adão José Cardoso”, Universidade Estadual de Campinas, Campinas, São Paulo, Brazil.

## Results

**Figures 5–17.**
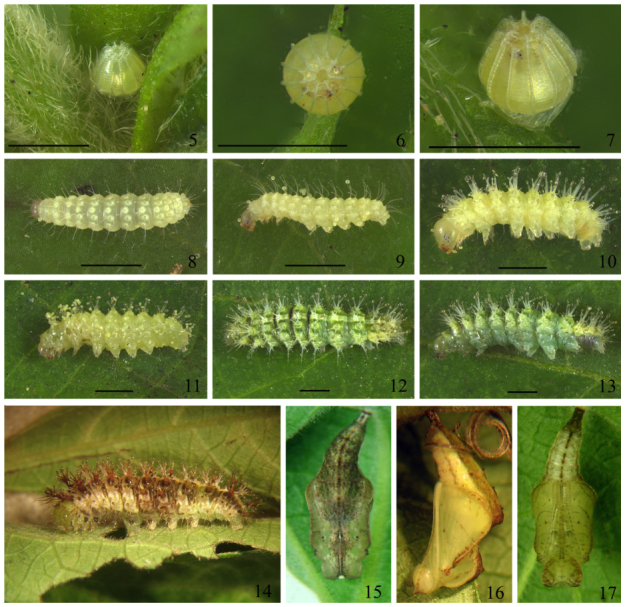
Dynamine *agacles agacles*. 5–7 Egg: (5) inside the pseudanthial inflorescence, (6) dorsal-superior view, (7) lateral view. 8–14 Larvae: (8, 9) 1^st^ instar, (8) dorsal view, (9) lateral view, (10) 2^nd^ instar in lateral view, (11) 3^rd^ instar in lateral view, (12, 13) 4^th^ instar, (12) dorsal view, (13) lateral view, (14) 5^th^ instar in lateral view. 15–17 Pupa: (15) dorsal view, (16) lateral view, (17) ventral view. Scale bar = 1 mm. High quality figures are available online.

**Egg** (Figures 5–7, 28, 29). Diameter 0.6 mm, height 0.5 mm (n = 14). Coloration uniformly greenish yellow; truncate, flatten in the base, with maximum diameter in the basal third, progressively narrowing toward the convex micropilar region in the apex; decorated with 14 vertical ribs forming prominent keels, and ca. 16–18 horizontal ribs visible only when crossing the vertical keels. Duration 7–8 days (n = 7).

**Figures 18–24.**
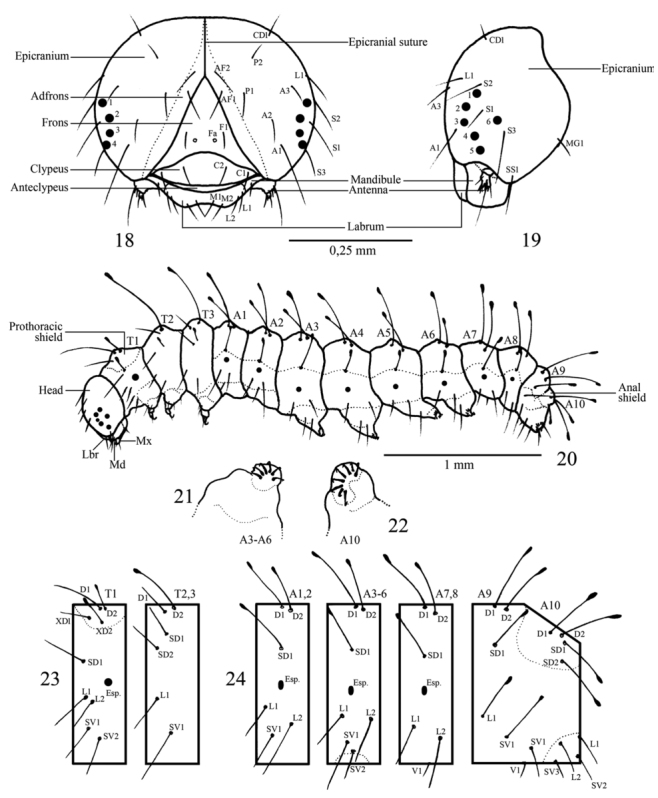
*Dynamine agacles agacles* 1^st^ instar: 18, 19 Head capsule: (18) frontal view, (19) lateral view. (20) lateral view of the larva. 21, 22 Crochets of the prolegs: (21) A3–A6, (22) A10. 23, 24 Body chaetotaxy: (23) thorax, (24) abdomen. Lbr — Labrum, Md — Mandibule, Mx — Maxilla.High quality figures are available online.

**First instar** (Figures 8, 9, 18–24, 30–37, 40, 43). Head capsule width 0.45 mm, height 0.38 mm (n = 3). Maximum body length 2.4 mm. Duration: 4 days (n = 10). Head pale brownish yellow, rounded and smooth, with pale yellow setae and without ornamentation; labrum bilobed; six dark stemmata disposed semicircularly; stemma 3 reduced. Body, prothoracic and anal shields pale yellow. Laterally projected spiracles with a smooth surface, rounded and bigger on the thorax while elliptical and smaller on the abdominal segments (Figures 36, 37). Legs and prolegs pale yellow; prolegs with uniordinal crochets (six in A3–A6 and eight in anal prolegs). Body setae light yellow and relatively long (ratio between setal length/segment height about 1.0), arising from chalazae; setae from subdorsal and dorsal series usually clubbed; the remaining pointed (see Figures 8, 9, 20, and 23); body covered with abundant microtrichia.

**Head chaetotaxy** (Figures 18, 19, 31–35). Consisted of 21 pairs of primary setae distributed in the following groups: clypeal C1 and C2; frontal group F1; adfrontal AF1 and AF2; anterior group A1, A2, A3; stemmatal S1, S2, S3; substemmatal SS1; microgenal MG1; lateral group L1; posteriodordal P1 and P2; cephalo—dorsal CD1 (following Duarte et al. 2005); labrum chaetotaxy L1, L2, M1 and M2. Pores not observed.

**Body chaetotaxy** (Figures 20, 23, 24, 30). Prothorax with nine pairs of setae, four on prothoracic shield (D1, D2, XD1, and XD2) D2 and XD2 clubbed, other pointed; SD1; the remaining distributed in two pinacula, the first with L1 and L2 and the second with SV1 with SV2. Mesothorax and metathorax with six pairs of setae, D1, D2, SD1, SD2, L1, and SV1, all pointed except D2, which is clubbed. Abdominal segments A1–A2 with six pairs of setae, D1, D2, SD1, L1, L2, and SV1; D1, D2, and SD1 clubbed, other pointed. Abdominal segments A3–A6 with seven pairs of setae, similar to A1–A2 with an additional SV2. Abdominal segments A7–A8 with six pairs of setae, D1, D2, SD1, L1, L2, and V1. Abdominal segments A9 with five pairs of setae, all clubbed, D1, D2, SD1, L1, and SV1. Abdominal segment A10 with ten pairs of setae, four on anal shield (D1, D2, SD1, and SD2) all clubbed; the remaining pointed (L1, L2, SV1, SV2, SV3, and V1).

**Second instar** (Figure 10). Head capsule width 0.52 mm; height 0.46 mm (n = 3). Maximum body length 4.0 mm. Duration: 3–4 days (n = 7). Head pale yellow, rounded and smooth, similar to previous instar; labrum and mandibles light brown. Body, prothoracic and anal shields pale yellow. Non—laterally projected spiracles, elliptical, all of them with similar size, this characteristic remains constant on the next three instars (Figures 38, 29). Legs and prolegs pale yellow; prolegs with biordinal crochets in mesopenellipse, very similar to those present in the third instar (Figures 41, 42).

**Third instar** (Figures 11, 38, 39, 41, 42). Head capsule width 0.74 mm; height 0.68 mm. Maximum body length 5.0 mm. Duration: 3–4 days (n = 7). In all similar to previous instar, but with a brownish yellow head.

**Fourth instar** (Figures 12, 13). Head capsule width 1.1 mm; height 0.96 mm. Maximum body length 8.0 mm. Duration: 3–5 days (n = 7). Head entire olive green, with epicranial suture, labrum and mandibles dark brown. Body entire green with prothoracic shield yellowish green; intersegmental region dorsally dark from T2–T3 to A3–A4; base of dorsal, subdorsal and lateral scoli yellowish green; a dorso—longitudinal pale yellow stripe from A1 to A8; ventral region pale green. All segments with short translucent setae pointed or clubbed. Anal shield olive green. Legs and prolegs pale yellowish green; prolegs A3–A6 with biordinal crochets in mesopenellipse; A10 with triordinal crochets in mesopenellipse.

**Fifth (last) instar** (Figure 14). Head capsule width 1.73 mm; height 1.6 mm. Maximum body length 12.0 mm. Duration: 4–6 days (n = 6). Head entire olive green, with dark brown epicranial suture, base of stemmata dark brown with a narrow cream stripe adjacent to each stemma. Body brownish cream laterally, greenish brown dorsally and ventrally, with dark intersegmental areas; prothoracic shield olive green with a narrow dorso—longitudinal cream stripe; a dorso—longitudinal cream stripe from T2 to A9; dorsal scolli reddish brown, the remaining cream. Anal shield olive green. Legs light brown, prolegs pale yellowish green; prolegs with triordinal crochets in mesopenellipse.

**Figures 25–27.**
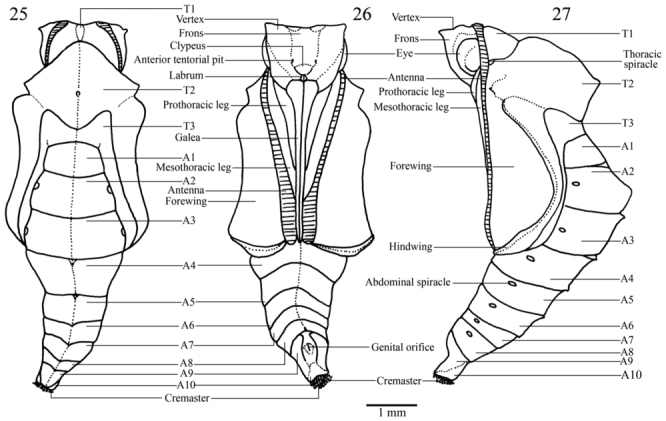
*Dynamine agacles agacles*. Dorsal, ventral and lateral view of the pupa. High quality figures are available online.

**Pupa** (Figures 15–17, 25–27, 44–49). 10 mm long. Duration 7 days (n = 6). Adecticous obtect, suspended by the cremaster. Initially dark green with light ventral region, changing to dark green or brown after one day, with a narrow cream dorso-longitudinal line from T1 to T3 and a narrow dark green ventral stripe from A4 to A8; profile elongated, with two conspicuous dorsal indentations, in T2 and A2, the abdominal indentation slightly forked. Head with two small projections on vertex; frons smooth; clypeus quadrangular; tentorial pit conspicuous dorso—lateral to the clypeus; labrum pentagonal and small; galea elongated from the labrum to the margin of the forewing cap; antennae with several transverse stripes from dorso—posterior head to distal galea; eye caps oval and lateral to anterior third of antenna, covering most of anterior head. Pronotum subrectangular and small; mesonotum large, with dorsal longitudinal ridge projecting as a point in the inferior portion; thoracic spiracle evident, lateral to the antenna in the T1–T2 intersegmental region; metanotum with antero—median margin convex. Prothoracic legs two—thirds the length of mesothoracic legs, arising adjacent to the anterior portion of eye cap and ending about half the galea length; mesothoracic legs lateral to the antennae, from the anterior third of the last until the posterior third of galea. Abdomen with antero—dorsal projections spined from A3 to A7; ellipsoidal spiracles from A2 to A8, the last two smaller than the others. Cremaster dark green, with simple dark brown distal hooks.

### Adult and larval behavior

In the three study sites, the only recorded host plant was *Dalechampia triphylla* Lam. (Malpighiales: Euphorbiaceae), a scandent vine common in clearings and forest edges.

Females were observed flying over only on plants bearing developed inflorescences. Females typically lay single eggs, in general on the involucral bract (either inside or outside the inflorescence), but eggs were also observed in other floral parts. Larvae eat part of the eggshell after hatching, and then feed preferentially on floral parts inside the inflorescences, including ovary, styles, pollen, the viscose resin, and even on the involucral bracts. Eggs were observed especially on flower buds, and the development of the larvae is closely linked with flower maturation, with no serious damage caused until the larva is fully—grown. At this time, the seeds are being formed, and the larva feeds on these until pupation.

In the first three instars, larvae remain inconspicuous nearby the floral parts, both due to their cryptic coloration and also due to pollen adherence in the setae and scolli tips. The greenish coloration after the fourth instar improves camouflaging with nearby fruits and seeds, where larvae usually rest in a semicircular posture.

In the laboratory, pupation was observed in secondary branches of *D. triphylla*, and pupae were observed suspended by the cremaster in a silk base made by the larva. Prepupa lasts about 24 hours, with larvae remaining suspended by the anal prolegs in a semicircular posture with head almost touching segment A6. Pupa remains motionless, but the abdominal segments are mobile, and can wriggle if the pupa is disturbed. Adults emerge through an opening in the dorsal region near the head, expelling a reddish brown meconium.

## Discussion

### Morphology of immature stages

The eggs of *D. agacles* are similar in morphology with those of most Biblidinae species, being truncate and with well—marked vertical ribs (Freitas and Oliveira 1992; Freitas et al. 1997; Greeney and Gerardo 2001). Most eggs of Biblidinae share this general pattern, with notable exceptions observed in the tribe Biblidini and Eurytelini (sensu Lamas 2004), which present unique “pilose” eggs (Johnston and Johnston 1980; Larsen 1991; Van Son 1979; Freitas and Brown Jr. 2008).

The first instar is characteristic within the Biblidinae in having relatively long body setae (ratio between setal length/segment height about 1.0), a feature also shared with all known Biblidini and Eurytelini, and with the genera *Cybdelis* Boisduval, 1836 and *Sea* Hayward, 1950, though the setae in the last two genera are somewhat shorter (ratio about 0.7) (Freitas et al. 1997). First instar of *D. agacles* has less than eight crochets on the prolegs; this character is shared with *D. postverta* (Cramer, 1779) (Freitas and Brown Jr. 2004). The only other Nymphalid genus observed with less than eight crochets on the prolegs of first instar is *Hypanartia* Hübner, (1821) (Nymphalinae) (Freitas and Brown Jr. 2004).

The absence of head scoli in later instars of *D. agacles* is a character also shared with other known *Dynamine* species (DeVries 1987). In all other Biblidinae, a pair of head scoli appears after the second instar (Muyshondt 1973; Muyshondt and Muyshondt 1975; Jenkins 1983, 1984, 1985a, 1985b, 1986, 1987, 1989, 1990; DeVries 1987; Freitas and Oliveira 1992; Neild 1996; Freitas et al. 1997; Greeney and Gerardo 2001; Freitas and Brown Jr. 2008; Barbosa et al. 2010). As described here, body scoli of *D. agacles* bear small viscose vesicles on the tip of the spines of the apical rosette. This unique structure of body scoli was first noted by Müller (1886) in *D. postverta*, and is also a possible synapomorphy of the genus *Dynamine*. However, the nature and function of this viscose substance is unknown and deserves further investigation.

### Natural history and evolution of *Dynamine*

All known host plants of *Dynamine* are species of Euphorbiaceae, especially in the genera *Tragia* and *Dalechampia*; records on *Sapium* (Euphorbiaceae) and *Canavalia* (Fabaceae) need confirmation (Beccaloni et al. 2008). Species of *Tragia* and *Dalechampia* are also host plants of the Neotropical genera *Biblis* Fabricius, 1807; *Mestra* Hübner, (1825); *Vila* Kirby, 1871; *Archimestra* Munroe, 1949; *Cybdelis* Boisduval, 1836; *Myscelia* Doubleday, (1845); *Hamadryas* Hübner, (1806) and *Ectima* Doubleday, (1848) (Beccaloni et al. 2008; Freitas and Brown Jr. 2008), and of the Paleotropical genera *Eurytela* Boisduval, 1833; *Byblia* Hübner, (1819); *Neptidopsis* Aurivillius, 1898 and *Ariadne* Horsfield, (1829) (Van Son 1979; Fontaine 1981; Wetherbee 1987a, 1987b; Teshirogi 1996). The habit of ovipositing and feeding of inflorescences appears to be a specialization of some *Dynamine* species, even if there are several species feeding on other plant parts (DeVries 1987; Neild 1996; Beccaloni et al. 2008). The behavior of ovipositing and feeding on inflorescences of *Dalechampia* was also reported for the Paleotropical Biblidinae *Neptidopsis ophione* (Cramer, 1779) (Armbruster and Mziray 1987), but the general lack of information on this species prevents us from further discussion in this issue.

Feeding on inflorescences probably resulted in various modifications of the larvae, deviating from the general Biblidinae pattern, such as the lack of head scoli, reduction in body scoli length, and sluglike appearance. All these traits are probably adaptations to facilitate movement and feeding inside buds and flower parts.

Additionally, the small size of most species of *Dynamine* (compared with remaining Biblidinae) could be related to use of a limiting larval resource (in this case inflorescences). As proposed by Thompson (1983), species feeding on small plant parts could be potentially smaller than their relatives, particularly those that feed internally on plant tissues being, therefore, unable to move easily between individual host plants; this hypothesis warrants investigation. By placing natural history information and morphological traits (including size) in a phylogenetic framework, we could attempt to understand the evolution of such life history traits in *Dynamine* butterflies in particular, and the association between body size and specialized feeding habits in general.

**Figures 28–33.**
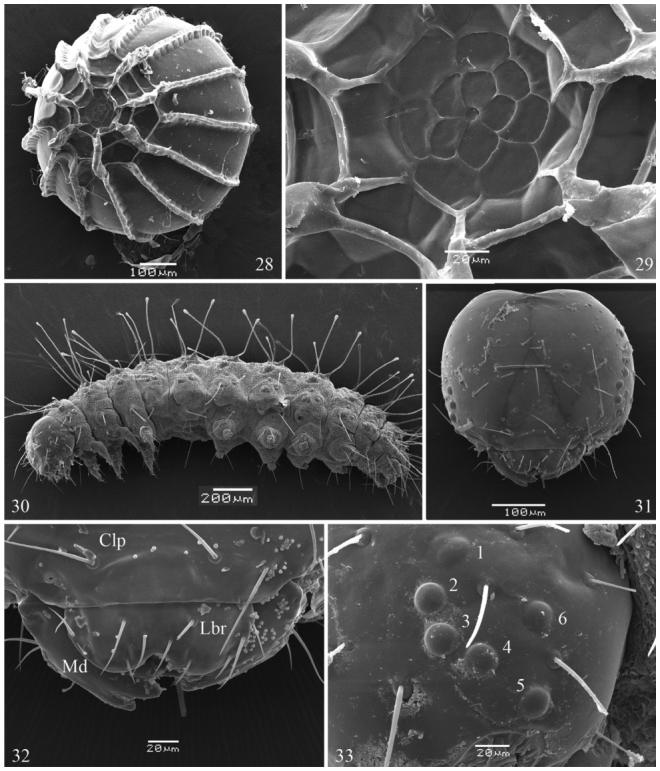
*Dynamine agacles agacles*. 28,29 Egg: (28) dorsalsuperior view, (29) micropilar region. 30–33 1^st^ instar larva: (30) lateral view, (31) head capsule in frontal view, (32) inferior region of the head, (33) stemmata region. Clp — Clypeus, Lbr — Labrum, Md — Mandibule. High quality figures are available online.

**Figures 34–39.**
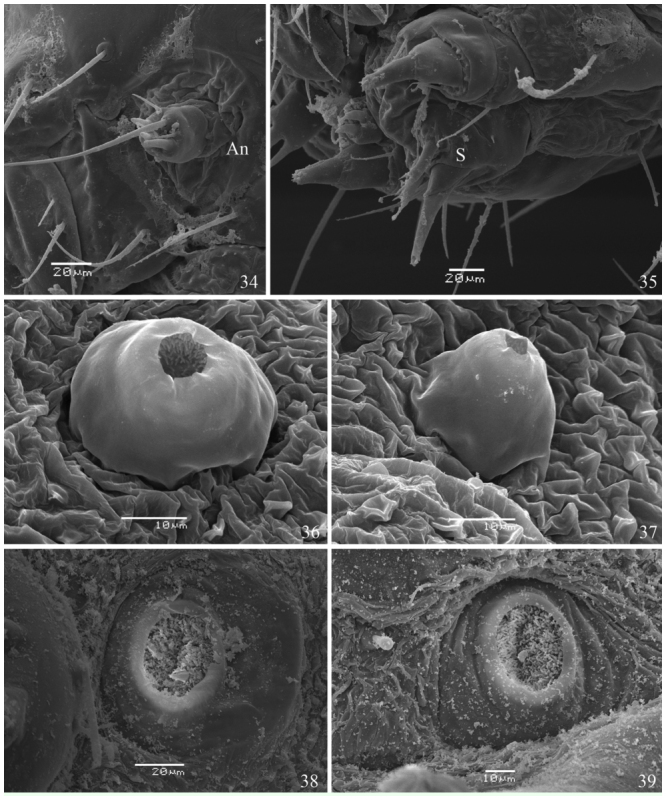
*Dynamine agacles agacles*. 34–37 1^st^ instar larva: (34) antenna, (35) spinneret, (36) thoracic spiracle, (37) abdominal spiracle. 38,39 3^rd^ instar larva: (38) thoracic spiracle, (39) abdominal spiracle. An — Antenna, S — Spinneret. High quality figures are available online.

**Figures 40–43.**
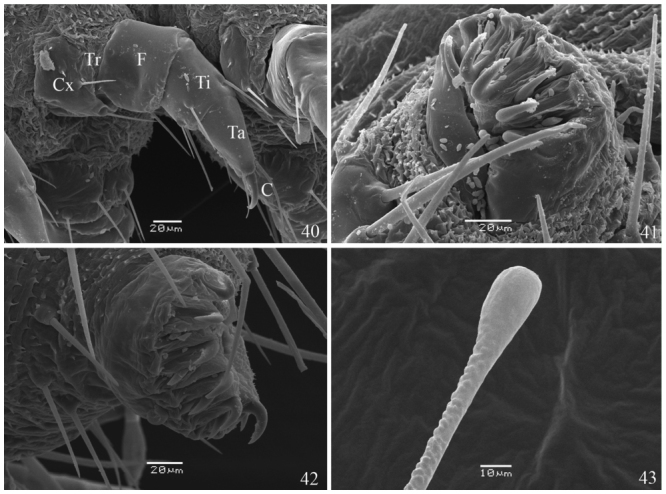
*Dynamine agacles agacles*. (40) thoracic leg of the 1^st^ instar larva. 41,42 3^rd^ instar larva: (41) A3 proleg, (42) A10 proleg. (43) dorsal scoli of the 1^st^ instar larva. C — Distal claw, Cx — Coxa, F — Femur, Ta — Tarsus, Ti — Tibia, Tr — Trochanter. High quality figures are available online.

**Figures 44–49.**
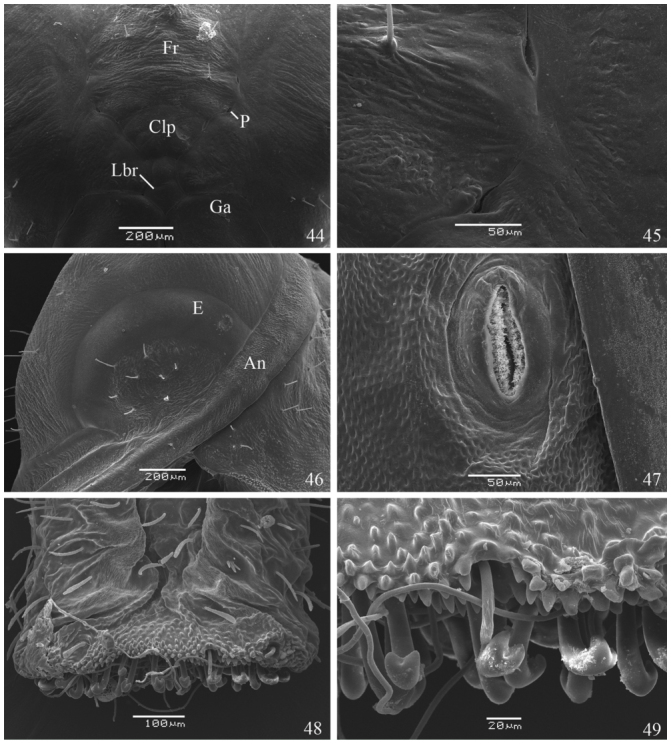
*Dynamine agacles agacles* Pupa: (44) ventral surface of the head, (45) detail of the anterior tentorial pit, (46) eye and part of the antenna, (47) abdominal spiracle, (48) A10 and ventral view of the cremaster, (49) detail of the cremaster hooks. An — Antenna, Clp — Clypeus, E — Eye, Fr — Frons, Ga — Galea, Lbr — Labrum, P — Anterior tentorial pit. High quality figures are available online.

## References

[bibr01] Ackery PR (1988). Host plants and classification: A review of nymphalid butterflies.. *Biological Journal of the Linnean Society*.

[bibr02] Armbruster WS, Mziray WR (1987). Pollination and herbivore ecology of African *Dalechampia* (Euphorbiaceae): comparisons with New World species.. *Biotropica*.

[bibr03] Barbosa EP, Kaminski LA, Freitas AVL (2010). Immature stages of the butterfly *Diaethria clymena janeira* (Lepidoptera: Nymphalidae: Biblidinae).. *Zoologia*.

[bibr04] Beccaloni GW, Hall SK, Viloria AL, Robinson GS (2008). *Catalogue of the hostplants of the Neotropical Butterflies/Catálogo de las plantas huéspedes de las mariposas Neotropicales*..

[bibr05] Brévignon C (2008). Notes sur Biblidinae, les Apaturinae et les Nymphalinae de Guyane Française (Lepidoptera: Nymphalidae).. *Lambillionea*.

[bibr06] Casagrande MM (1979). Sobre *Caligo beltrao* (Illiger). I. Taxonomia, biologia, morfologia das fases imaturas e distribuições espacial e temporal (Lepidoptera, Satyridae, Brassolinae).. *Revista Brasileira de Biologia*.

[bibr07] Casagrande MM, Mielke OHH (2005). Larva de quinto estádio e pupa de *Opsiphanes quiteria meridionalis* Staudinger (Lepidoptera, Nymphalidae, Brassolinae).. *Revista Brasileira de Entomologia*.

[bibr08] DeVries P. (1987). *The Butterflies of Costa Rica and their natural history: Papilionidae, Pieridae, and Nymphalidae*..

[bibr09] Dias MM, Costa C, Ide S, Simonka CE (2006). Lepidoptera.. *Insetos Imaturos. Metamorfose e Identificação*.

[bibr10] Dias FMS, Casagrande MM, Mielke OHH (2010a). Aspectos biológicos e morfologia externa dos imaturos de *Memphis moruus stheno* (Prittwitz) (Lepidoptera: Nymphalidae).. *Neotropical Entomology*.

[bibr11] Dias FMS, Casagrande MM, Mielke OHH (2010b). Biology and external morphology of immature stages of *Memphis appias* (Hübner) (Lepidoptera: Nymphalidae: Charaxinae).. *Zootaxa*.

[bibr12] Doyle JF (1979). Temporary range extension and larval foodplant of *Dynamine dyonis* (Nymphalidae) in Texas.. *Journal of the Lepidopterists' Society*.

[bibr13] Duarte M, Robbins RK, Mielke OHH (2005). Immature Stages of *Calycopis caulonia* (Hewitson, 1877) (Lepidoptera, Lycaenidae, Theclinae, Eumaeini), with notes on rearing detritivorous hairstreaks on artificial diet.. *Zootaxa*.

[bibr14] Fontaine M. (1981). Lép.: Nymphalidae Sousfam. Eurytelinae. Premiers états des espèces observées á Isiro (ex. Paulis) et environs immédiats. Zaire: region du Haut-Zaire, sous region du Haut-Uele.. *Lambillionea*.

[bibr15] Freitas AVL, Oliveira PS (1992). Biology and behavior of the Neotropical butterfly *Eunica bechina* (Nymphalidae) with special reference to larval defense against ant predation.. *Journal of Research on the Lepidoptera*.

[bibr16] Freitas AVL, Brown KS, Otero LD (1997). Juvenile stages of *Cybdelis*, a key genus uniting the diverse branches of the Eurytelinae.. *Tropical Lepidoptera*.

[bibr17] Freitas AVL, Brown KS (2004). Phylogeny of the Nymphalidae (Lepidoptera).. *Systematic Biology*.

[bibr18] Freitas AVL (2007). A new species of *Moneuptychia Forster* (Lepidoptera: Satyrinae: Euptychiina) from the highlands of Southeastern Brazil.. *Neotropical Entomology*.

[bibr19] Freitas AVL, Brown KS (2008). Immature stages of *Vila emilia* (Lepidoptera: Nymphalidae, Biblidinae).. *Tropical Lepidoptera*.

[bibr20] Greeney HF, Gerardo NM (2001). Descriptions of the Immature Stages and Oviposition Behavior of *Pyrrhogyra otolais* (Nymphalidae).. *Journal of the Lepidopterists' Society*.

[bibr21] Hasenfuss I, Kristensen NP, Kristensen NP (2003). Skeleton and muscles: Immatures. *Lepidoptera: Moths and butterflies 2. Handbuch der Zoologie / Handbook of Zoology* IV/36..

[bibr22] Jenkins DW (1983). Neotropical Nymphalidae. I. Revision of *Hamadryas*.. *Bulletin of the Allyn Museum*.

[bibr23] Jenkins DW (1984). Neotropical Nymphalidae. II. Revision of *Myscelia*.. *Bulletin of the Allyn Museum*.

[bibr24] Jenkins DW (1985a). Neotropical Nymphalidae. III. Revision of *Catonephele*.. *Bulletin of the Allyn Museum*.

[bibr25] Jenkins DW (1985b). Neotropical Nymphalidae. IV. Revision of *Ectima*.. *Bulletin of the Allyn Museum*.

[bibr26] Jenkins DW (1986). Neotropical Nymphalidae. V. Revision of *Epiphile*.. *Bulletin of the Allyn Museum*.

[bibr27] Jenkins DW (1987). Neotropical Nymphalidae. VI. Revision of *Asterope* (= *Callithea* Auct).. *Bulletin of the Allyn Museum*.

[bibr28] Jenkins DW (1989). Neotropical Nymphalidae. VII. Revision of *Nessaea*.. *Bulletin of the Allyn Museum*.

[bibr29] Jenkins DW (1990). Neotropical Nymphalidae. VIII. Revision of *Eunica*.. *Bulletin of the Allyn Museum*.

[bibr30] Johnston G, Johnston B (1980). *This is Hong Kong: Butterflies*..

[bibr31] Kaminski LA, Dell'Erba R, Moreira GRP (2008). Morfologia externa dos estágios imaturos de Heliconíneos Neotropicais: VI. *Dione moneta moneta* Hübner (Lepidoptera, Nymphalidae, Heliconiinae).. *Revista Brasileira de Entomologia*.

[bibr32] Larsen TB (1991). *The Butterflies of Kenya and Their Natural History*..

[bibr33] Lamas G, Heppner JB (2004). Checklist: part 4A. Hesperioidea—Papilionoidea.. *Atlas of Neotropical Lepidoptera*.

[bibr34] Mosher E (1916). A classification of the Lepidoptera based on characters of the pupa.. *Bulletin of the Illinois State Laboratory of Natural History*.

[bibr35] Müller W (1886). Sudamerikanische Nymphalidenraupen. Versuch eines natürlichen systems der Nymphaliden.. *Zoologische Jahrbuecher (Jena)*.

[bibr36] Munroe E (1949). A new genus of Nymphalidae and its affinities (Lepidoptera, Rhopalocera).. *Journal of the New York Entomological Society*.

[bibr37] Muyshondt A (1973). Notes on the life cycle and natural history of butterflies of E1 Salvador. I A.— *Catonephele numilia esite* (Nymphalidae-Catonephelinae).. *Journal of the New York Entomological Society*.

[bibr38] Muyshondt A, Muyshondt A (1975). Notes on the life cycle and natural history of butterflies of El Salvador. II B.- *Hamadryas guatemalena* Bates (NymphalidaeHamadryadinae).. *Journal of the New York Entomological Society*.

[bibr39] Neild AFE (1996). *The Butterflies of Venezuela. Part I: Nymphalidae (Limenitidinae, Apaturinae, Charaxinae)*..

[bibr40] Paim AC, Kaminski LA, Moreira GRP (2004). Morfologia externa dos estágios imaturos de Heliconíneos Neotropicais : IV. *Dryas iulia alcionea* (Lepidoptera, Nymphalidae, Heliconiinae).??? *Iheringia*.. Série Zoologia.

[bibr41] Peterson A (1962). *Larvae of Insects. An Introduction to Neartic Species. Part I Lepidoptera and Plant Infesting Hymenoptera*..

[bibr42] Scoble MJ (1992). *The Lepidoptera: form, function and diversity*..

[bibr43] Souza NA, Veiga AFSL, Casagrande MM, Godim MGC (2006). Morfologia externa dos imaturos de *Caligo teucer* (Linnaeus, 1758) (Lepidoptera, Nymphalidae).. *Revista Brasileira de Zoologia*.

[bibr44] Stehr FW, Stehr FW (1987). Order Lepidoptera.. *Immature Insects*..

[bibr45] Teshirogi M (1996). *An illustrated book of the Japanese Nymphalidae*..

[bibr46] Thompson JN (1983). Selection pressures on phytophagous insects feeding on small host plants.. *Oikos*.

[bibr47] Van Son D (1979). *The butterflies of Southern Africa. Part IV. Nymphalidae: Nymphalinae*..

[bibr48] Wetherbee DK (1987a). *Life—stages of Archimestra teleboas and Dynamine egaea (Nymphalidae, Papilionoidea)*..

[bibr49] Wetherbee DK (1987b). *Life-stages of Hamadryas amphicloe diasia in Hispaniola (Rhopalocera, Nymphalidae)*..

[bibr50] Willmott KR, Hall JPW (2010). A new species of *Dynamine* Hübner, (1819) from northwestern Ecuador (Lepidoptera: Nymphalidae: Biblidinae).. *Tropical Lepidoptera Research*.

